# Certificates of confidentiality and unexpected complications for pragmatic clinical trials

**DOI:** 10.1002/lrh2.10238

**Published:** 2020-07-14

**Authors:** Jeremy Sugarman, Judith Carrithers

**Affiliations:** ^1^ Berman Institute of Bioethics Johns Hopkins University Baltimore Maryland USA; ^2^ School of Medicine Johns Hopkins University Baltimore Maryland USA; ^3^ Bloomberg School of Public Health (JS) Johns Hopkins University Baltimore Maryland USA; ^4^ Advarra Columbia Maryland USA

**Keywords:** confidentiality, policy, pragmatic clinical trials, privacy, research ethics

## Abstract

**Introduction:**

The need to protect the confidentiality of research data has long been recognized. One means to help protect research data from use in civil or criminal matters in the United States is a Certificate of Confidentiality (CoC). Until recently, investigators applied for a CoC when conducting research that was sensitive, stigmatizing or where the disclosure of private information could possibly result in civil or criminal liability. However, effective October 1, 2017, CoCs are automatically issued for much research supported by the National Institutes of Health (NIH). While automatic issuance reduces administrative burden, it also poses some surprising unanticipated challenges for research in general and pragmatic clinical trials (PCTs) in particular, which are key elements of learning health systems.

**Methods:**

We reviewed the new policy on CoCs to identify and analyze issues related to it that are potentially problematic for PCTs.

**Results:**

We identified three relevant issues: (1) whether the EHR may be populated with research data that may be sensitive or stigmatizing without explicit consent from subjects; (2) incomplete protections for sensitive data in the EHR; and (3) requirements for notifying subjects about the CoC provisions.

**Conclusion:**

Formal guidance from the NIH is needed to address the application of CoCs to the setting of PCTs. In the meantime, it is essential for researchers designing and conducting PCTs, as well as health care systems in which this research is conducted, to be aware of the nuances inherent in CoCs so they can best adhere to their legal obligations regarding them. In the absence of guidance, special attention should be paid to pragmatic research that populates the electronic health record with research data as well as research conducted without explicit consent. Given the large amount of pragmatic research precipitated by the Coronavirus Disease 2019 pandemic, which has been accompanied by major efforts to share data, the need for such guidance is especially urgent.

## 1 INTRODUCTION

The need to protect the confidentiality of research data, particularly sensitive or stigmatizing research data, has long been recognized, not only as an ethical obligation to protect research participants, but also as instrumental to conducting research. Simply put, if those eligible to participate are not assured of data protection they may be unwilling to enroll in research or reluctant to reveal sensitive information essential to the research. One means that has been utilized to help protect such research data from use in civil or criminal matters in the United States is a Certificate of Confidentiality (CoC).^1^ Until recently, investigators applied to the National Institutes of Health (NIH) for a CoC only when conducting research that was sensitive, stigmatizing or where the disclosure of private information could possibly result in civil or criminal liability.^2^ However, effective October 1, 2017, pursuant to the 21st Century Cures Act^3^ that aims to accelerate research, CoCs are automatically issued for all NIH funded research within the scope of the new policy, which is both broader than the prior policy and redefines identifiable data.^4^, ^5^, ^6^ While the automatic issuance of a CoC reduces administrative burden, it also poses some surprising unanticipated challenges for research in general^6^ and pragmatic clinical trials (PCTs) and comparative effectiveness research in particular, which are key elements of learning health systems.

PCTs are being increasingly used to generate evidence to guide healthcare decision‐making by patients, clinicians and payers. By harnessing data already available in electronic health records (EHRs) and involving larger and more diverse populations, PCTs not only reduce research costs but are also positioned to generate generalizable findings. The CoC policy seems designed for traditional clinical research where research interventions are distinct from routine clinical care and research records are separate from clinical records. In pragmatic research, the focus is on embedding the research interventions in clinical care and the research relies as much as possible on existing clinical records. Dissolving those boundaries is valuable in answering important research questions, but raises a variety of ethics and regulatory issues, including those related to the new CoC policy described here.

Like all research, responsibly designing and conducting PCTs necessitates identifying and addressing a range of ethical and regulatory issues to help ensure that the rights, interests and welfare of those who are involved are protected.^7^ For example, since many PCTs evaluate standards of care, the traditional informed consent process is often modified in various ways (eg, waiver of consent, opt‐out notification, oral consent). However, it can be difficult to determine when it is appropriate to use such alternatives.^8^ In addition, while PCTs do not typically entail additional burdens for research subjects, there can be particular challenges related to ensuring the privacy and confidentiality of subjects.^9^ PCTs often include the use of clinical data across different health systems, which heightens concerns about data privacy and confidentiality, particularly when sensitive information is being collected (eg, illicit drug use, sexually transmitted infections). For example, this could include pragmatic research aimed at addressing major public health issues such as HIV and the opioid crisis.^10^ While at first glance a CoC might seem well suited to help manage such concerns, the current CoC provisions without guidance or modification present challenges for many PCTs.

In this paper, after outlining the provisions of the new CoC policy, we describe selected issues that seem especially problematic in the context of PCTs and thereby threaten learning health systems.

## 2 KEY PROVISIONS OF THE CoC POLICY

As noted earlier, NIH funded or conducted human subjects research is now issued a CoC automatically, which makes the scope of research covered by the policy much broader. The new policy applies to all biomedical, behavioral, clinical or other research funded wholly or in part by the NIH that “collects or uses identifiable sensitive information”.^4^ Identifiable sensitive information is defined to include information about an individual that is “gathered or used during” the research and (1) the individual is identified, or (2) where “there is at least a very small risk, that some combination of the information, a request for information and other available data sources could be used to deduce the identity of the individual”.^4^ The policy now covers research deemed exempt under federal regulations unless the information obtained is recorded in such a manner that human subjects cannot be identified or the identity of the human subjects cannot readily be ascertained, directly or through identifiers linked to the subjects; biospecimen research where the identity of the source might be ascertained by using available data sources; the generation of individual level human genomic data from biospecimens or the use of such data; and any other human subjects research that involves information that might identify an individual.^4^ Researchers bear the responsibility for determining if their research falls under the policy and if so, specific provisions regarding permissible disclosures apply (see Table 1). In addition, researchers must “ensure that any investigator or institution not funded by NIH who receives a copy of identifiable, sensitive information protected by a Certificate issued by this Policy, understand they are also subject to the requirements”.^4^ Finally, “For studies in which informed consent is sought, NIH expects investigators to inform research participants of the protections and the limits to protections provided by a Certificate.”^4^

**TABLE 1:** Disclosure requirements under NIH CoC policy^4^

"[T]he recipient of the Certificate shall not: Disclose or provide, in any Federal, State, or local civil, criminal, administrative, legislative, or other proceeding, the name of such individual or any such information, document, or biospecimen that contains identifiable, sensitive information about the individual and that was created or compiled for purposes of the research, unless such disclosure or use is made with the consent of the individual to whom the information, document, or biospecimen pertains; or Disclose or provide to any other person not connected with the research the name of such an individual or any information, document, or biospecimen that contains identifiable, sensitive information about such an individual and that was created or compiled for purposes of the research. Disclosure is permitted only when: Required by Federal, State, or local laws (eg, as required by the Federal Food, Drug, and Cosmetic Act, or state laws requiring the reporting of communicable diseases to State and local health departments), excluding instances of disclosure in any Federal, State, or local civil, criminal, administrative, legislative, or other proceeding; Necessary for the medical treatment of the individual to whom the information, document, or biospecimen pertains and made with the consent of such individual; Made with the consent of the individual to whom the information, document, or biospecimen pertains; or Made for the purposes of other scientific research that is in compliance with applicable Federal regulations governing the protection of human subjects in research."

## 3 SELECTED ISSUES FOR PCTs

Three issues regarding the new policy on CoCs are particularly problematic in the setting of PCTs: (1) whether the EHR may be populated with research data that may be sensitive or stigmatizing without explicit consent from subjects; (2) incomplete protections for sensitive data in the EHR; and (3) requirements for notifying subjects about the CoC provisions.

### 3.1 The permissibility of populating the EHR with sensitive data

Institutional policy may dictate whether research data can be included in the EHR. Although this policy may apply to all research, it may impact PCTs more directly as they commonly integrate study interventions with other mental health and clinical care and include the research data in the EHR. Depending on the research, the research data may include sensitive information covered under the CoC. Once this information is included in the EHR, it is likely to be accessible to a variety of people, including clinicians and others with legitimate access to medical records. As shown in Table 1, under a CoC an investigator is not permitted to disclose or provide to others not connected with the research the name of a research participant or other information, documents or biospecimens that contain identifiable, sensitive information about the research participant that was created or compiled for purposes of the research, unless such disclosure or use is made with the consent of the subject (or as otherwise required by law or regulation).^4^

For PCTs conducted with explicit consent, the consent form would typically explain that research data will be included in the EHR and by signing the consent form the research participant agrees to disclosures of research information from the EHR. However, many PCTs are conducted under a modification or waiver of consent that has been approved by an Institutional Review Board (IRB).^8^ In these studies, the research data would be populated into the EHR without the subject's consent. Based on the current CoC disclosure requirements, it is not clear whether including identifiable data in the EHR and allowing disclosure of that data outside of the research without consent is permitted.^6^

The NIH CoC Frequently Asked Questions includes the following, which appears to leave it to the site to determine the appropriateness of including research data in the EHR without consent, and does not address how the CoC protections and disclosure requirements apply in this situation.

14. As with many pragmatic trials, my study is fully integrated with clinical care, such that for research purposes we will extract information from the medical records and for clinical care we intend to incorporate research data into the medical records. Must we have the participants' consent to put research data in the medical records?

The policies for handling research data and medical records can differ with each institution. For this reason, NIH suggests that investigators who intend to include research data in subjects' medical records work with their own institutional counsel and IRB to ensure that all documents are handled appropriately and in line with the institution's own policies.^11^

### 3.2 Incomplete protections for sensitive data in the EHR

The CoC protects “names or any information, documents, or biospecimens containing identifiable, sensitive information related to a research participant”.^1^ If separate research records are maintained for the study, the CoC protections could be implemented. However, once this information is included in the EHR, the question of whether the CoC protections apply and can be implemented becomes more complex.^6^ Perhaps paradoxically, a CoC provides incomplete protection of sensitive data because the EHR is unlikely to be deemed a research record and the CoC protections and investigator responsibilities apply only to the research records. Nevertheless, research data incorporated in the EHR arguably warrant similar protections. Although these protections are important for all research data, they become even more critical when the data are generated through a research activity where subjects were enrolled with a waiver of consent and were not aware of the research.

### 3.3 Requirements for notifying subjects about CoCs

The CoC policy requires that participants be notified of the CoC protections and limitations for research “in which informed consent is sought”. While this requirement is clear for conventional research that involves explicit written informed consent, and NIH offers template language to do so,^12^ it is less clear for PCTs that may use a wide array of alternative approaches to providing information about the research and seeking permission to participate.^8^ These approaches may include no disclosure (waiver of consent), simple disclosure with opt‐out or opt‐in provisions (alteration of consent), oral consent, and a brief or standard written consent process. Each of these approaches can comport with federal research regulations and be approved by an IRB. However, it is unclear if the requirement to notify participants about a CoC extends to these approaches. For studies conducted with a waiver of consent, there is no mechanism for notifying participants of the CoC provisions. For studies conducted with simple disclosure or abbreviated processes of consent and authorization, notification of the CoC protections could be burdensome, pose barriers to research efficiency, perhaps inflate the importance of the information compared to other information that is not being disclosed and affect research design. However, disclosing this information provides transparency and may promote trust in the research enterprise.

Given the varying approaches to consent in PCTs and the integration of research data from PCTs in the EHR, guidance regarding what counts as “informed consent” under the CoC policy is needed.

## 4 CONCLUDING COMMENTS

While the NIH policy regarding CoCs derives from the 21st Century Cures Act and therefore must adhere to its stipulations unless Congress modifies it, formal guidance from the NIH is needed to address the application of CoCs to the setting of PCTs. In the meantime, it is essential for researchers designing and conducting PCTs, as well as health care systems in which this research is conducted, to be aware of the nuances inherent in CoCs so they can best adhere to their legal obligations regarding them.^6^ In the absence of guidance, special attention should be paid to pragmatic research that populates the EHR with research data as well as research conducted without explicit consent.

Finally, the unprecedented Coronavirus Disease 2019 (COVID‐19) public health emergency obviously necessitates a broad array of research efforts aimed at identifying safe and effective means of prevention and treatment, including explanatory and pragmatic research. When such research is supported by the NIH, the CoC provisions would apply and researchers must be sensitive to them. While it is beyond the scope of this article to examine COVID‐19 pandemic research efforts and the potential issues that may arise in regard to CoCs for them in detail, special challenges may arise as investigators are prompted to participate in enhanced data sharing to accelerate scientific understanding as a means to help attenuate the pandemic.^13^ This makes the need for formal guidance about CoCs from the NIH especially urgent.

## CONFLICT OF INTEREST

Jeremy Sugarman is a member of Merck KGaA's Bioethics Advisory Panel and Stem Cell Research Oversight Committee; he is a member of IQVIA's Ethics Advisory Panel; is a member of the Scientific Advisory Board for Aspen Neurosciences; and he has consulted for Portola Pharmaceuticals, Inc. None of these relationships are related to the material discussed in this manuscript. Judith Carrithers is the Director of Regulatory Affairs at an independent institutional review board (Advarra), which also provides research consulting services.

## AUTHOR CONTRIBUTIONS

Both authors contributed to the conception, drafting and critical revision of the paper. All authors approved the final version of the paper.
